# Use of veno-venous extracorporeal membrane oxygenation for stabilization prior to redo sternotomy for aortic pseudoaneurysm repair

**DOI:** 10.21542/gcsp.2024.6

**Published:** 2024-01-03

**Authors:** Anson Y. Lee, Emily L. Larson, Ifeanyi D. Chinedozi, Jennifer S. Lawton, Hamza Aziz

**Affiliations:** 1 University of Hawaii, John A. Burns School of Medicine, Honolulu, HI, USA; 2 Division of Cardiac Surgery, Department of Surgery, Johns Hopkins University School of Medicine, Baltimore, MD, USA

## Abstract

**Background:** Aortic pseudoaneurysms are particularly dangerous because of the risk of rupture and compression of mediastinal structures, including the trachea, and resultant respiratory distress. If respiratory distress progresses to respiratory failure, extracorporeal membrane oxygenation may be used to provide oxygenation prior to or during pseudoaneurysm repair.

**Case presentation:** A 62-year-old male with a history of emergent aortic ascending and arch replacement for Stanford Type A dissection 10 months prior presented to his primary care physician with dyspnea. Chest radiography revealed a widened mediastinum, and subsequent computed tomography angiogram revealed a pseudoaneurysm at the distal suture line of the aortic arch replacement. Due to the location of the pseudoaneurysm, the patient’s trachea was compressed, and he was emergently placed on veno-venous (VV) extracorporeal membrane oxygenation (ECMO) following unsuccessful intubation for respiratory distress. Two days later, the patient underwent a redo sternotomy and repair of a 2–3 mm defect in the anterior aspect of the distal suture line of the prior aortic arch replacement. The patient progressed well and was discharged on postoperative day 13.

**What we learned**: Using a combination of peripheral bypass, hypothermic circulatory arrest, delayed closure, and respiratory support, this case demonstrates how even complex patients can be successfully treated with multiple strategies.

## Introduction

Pseudoaneurysms are locally formed hematomas secondary to arterial injury contained by products of the coagulation cascade and do not involve the vessel wall. In contrast, true aneurysms are outpouchings contained by all three layers of the arterial wall^1^. Pseudoaneurysms are commonly associated with catheter-based procedures that can decrease the integrity of the arterial wall, but may also form at the suture lines of arterial repairs^2–8^. Iatrogenic femoral pseudoaneurysms following femoral catheterization are most commonly reported, with aortic pseudoaneurysms being less common^9–12^. Because of this rarity, reports on aortic pseudoaneurysms are primarily limited to case reports and series^13–24^. Patel et al. published a review of multiple single-institution studies in 2014 that specifically analyzed the role of percutaneous closure in these patients. They found that, although percutaneous closure provided satisfactory short-term outcomes, there were associated risks of embolization, device migration, and leaks^25^. For patients in whom percutaneous closure is not an option, endovascular graft placement or open repair remains as an alternative^3,18^.

Aortic pseudoaneurysms pose an additional unique risk of tracheal compression owing to their anatomical location. Thus, respiratory distress and even failure can occur depending on the size and location of the aneurysm. If endotracheal intubation is not possible due to the anatomical location of such aneurysms, ECMO may be required to provide adequate oxygenation^26,27^. However, ECMO is associated with additional operative risks, which may be a barrier to its implementation^28–31^. To the best of our knowledge, no study has reported aortic pseudoaneurysm repair in patients receiving ECMO.

### Case report

A 62-year-old male underwent aortic ascending and arch replacement for emergent Stanford Type A dissection 10 months prior to presentation. Additional relevant surgical history included Stanford Type B dissection repair, Crawford type III thoracoabdominal aneurysm repair, abdominal aortic aneurysm repair, and atrial septal defect repair *via* sternotomy. The medical history included essential hypertension, pulmonary hypertension, chronic diastolic heart failure, and chronic kidney disease.

In the most recent procedure, Stanford Type A dissection began at the ascending aorta, extended into the arch along the lesser curvature, and did not involve the arch vessels. A graft was placed with the distal anastomosis to the proximal descending aorta 1–2 cm proximal to the prior thoracoabdominal graft, with some sections of this anastomosis consisting of a graft-to-graft anastomosis. The proximal anastomosis was two centimeters above the right coronary artery. Additionally, the arch vessels were isolated as island grafts and attached to the arch graft. The patient did well and was discharged on aspirin, atorvastatin, and labetalol.

**Figure 1.**
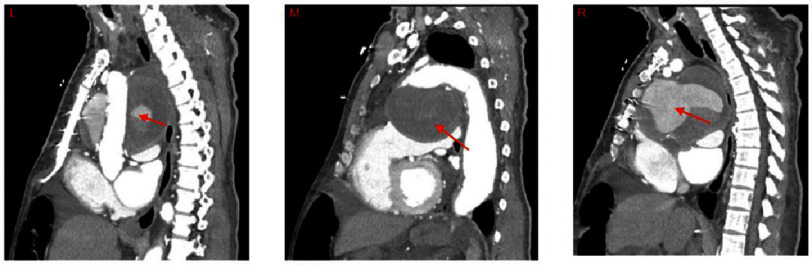
Sagittal Images of CT scan of a patient on VV ECMO with an aortic pseudoaneurysm at the distal suture line of a prior aortic arch replacement. Left: Large pseudoaneurysm (arrow) behind the aortic graft, compressing the trachea and esophagus posteriorly. Middle: Large pseudoaneurysm below arch (arrow), compressing heart inferiorly. Right: Large pseudoaneurysm (arrow) just posterior to the sternum, compressing the heart inferiorly while compressing the esophagus and trachea posteriorly. Abbreviations: CT - computed tomography, CTA - computed tomography angiogram, VV ECMO - venovenous extracorporeal membrane oxygenation.

**Figure 2.**
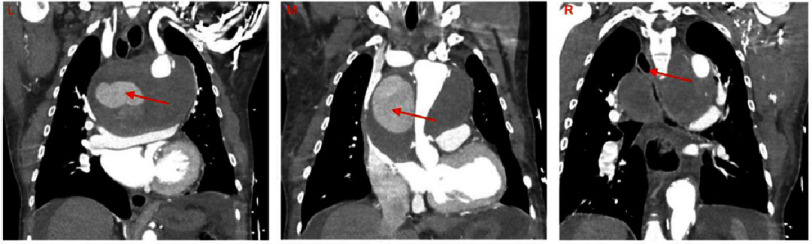
Coronal Images of CT scan of a patient on VV ECMO with an aortic pseudoaneurysm at the distal suture line of a prior aortic arch replacement. Left: Large pseudoaneurysm (arrow) with graft anastomosis visible just distal to the left subclavian, left atrium and heart compressed inferiorly. Middle: Large pseudoaneurysm (arrow) leading to compression of the aortic graft. Right: Large pseudoaneurysm compressing the trachea (arrow) with rightward displacement. Abbreviations: CT - computed tomography, CTA - computed tomography angiogram, VV ECMO - veno-venous extracorporeal membrane oxygenation.

**Figure 3.**
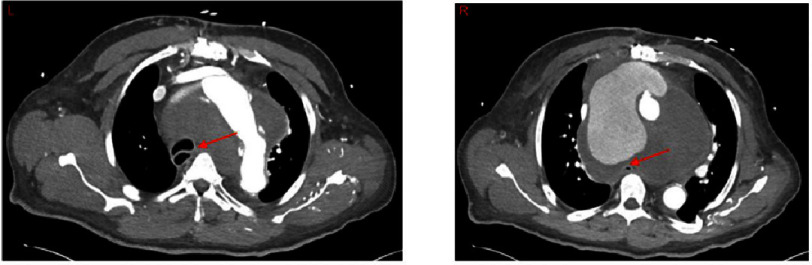
Axial Images of CT scan of a patient on VV ECMO with an aortic pseudoaneurysm at the distal suture line of a prior aortic arch replacement. Left: Aortic arch with large pseudoaneurysm and compression and distortion of the trachea (arrow) to the right side. Right: Aortic extravasation of contrast into pseudoaneurysm which can be seen compressing the esophagus (arrow). Abbreviations: CT - computed tomography, CTA - computed tomography angiogram, VV ECMO - venovenous extracorporeal membrane oxygenation.

Ten months later, the patient presented to his primary care physician after experiencing dyspnea for several months. An initial chest X-ray scan revealed a widened mediastinum. Three days later, the patient presented to the emergency department with acutely worsening dyspnea. A computed tomography angiogram (CTA) revealed a pseudoaneurysm at the distal suture line of the aortic arch graft from the prior acute Type A aortic dissection repair which could be viewed in the sagittal (Figure 1), coronal (Figure 2), and axial (Figure 3) planes. The pseudoaneurysm distorted the mediastinal anatomy and compressed the trachea. The aortic pseudoaneurysm also abutted the posterior sternal table. The patient was transferred to the intensive care unit, where he was placed on bi-level positive airway pressure (BiPAP) for poor oxygenation. Over the next 16 h, the patient became more fatigued as PCO2 increased to 87 mmHg. Additionally, he desaturated and became increasingly tachypneic and diaphoretic. Endotracheal intubation was attempted for acute hypoxic respiratory failure, but was unsuccessful. The decision was made to perform emergent venovenous extracorporeal membrane oxygenation (VV ECMO) *via* bilateral femoral vein cannulation. ECMO flows of 3.2 L/min were achieved with patient stabilization and improved oxygenation.

The following day, the patient required cannula adjustment due to poor flow as low as 2 L/min and PaO2 of 33 mmHg. The inflow and outflow cannulas were adjusted, with subsequent flow improvement to 4 L/min.

**Figure 4.**
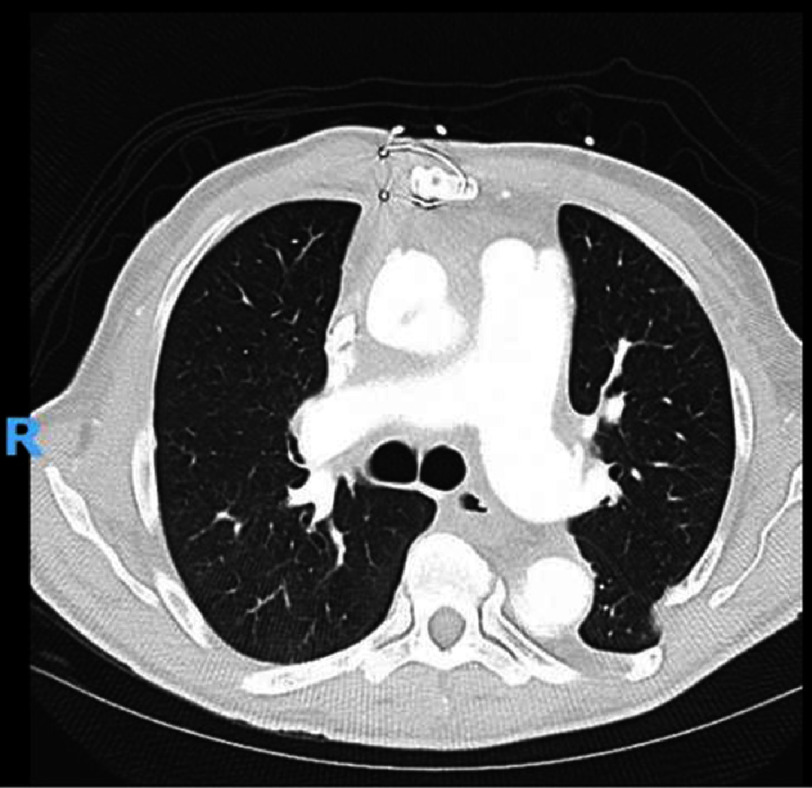
Axial Image of a postoperative CT scan of a patient following the repair of an aortic pseudoaneurysm at the distal suture line of a prior aortic arch replacement on VV ECMO. Axial image of the chest at the level of T7 in a patient following pseudoaneurysm repair 10 months after ascending and aortic arch replacement for acute type A aortic dissection. Mediastinal anatomy and trachea are consistent with resolution of the large pseudoaneurysm. Abbreviations: CT is computed tomography, VV ECMO is venovenous extracorporeal membrane oxygenation.

The following day, the patient underwent a redo sternotomy and pseudoaneurysm repair. Because the preoperative CT scan of the pseudoaneurysm revealed it was abutting the posterior sternal table, VV ECMO was converted to cardiopulmonary bypass using a peripheral arterial cannula in the right femoral artery. Upon entry into the chest, no identifiable cardiac structures were observed, leading to difficulty in dissecting the right heart border. The decision was made to use deep hypothermic circulatory arrest (HCA) to repair the pseudoaneurysm. After the initiation of HCA (18 °C), the pseudoaneurysm was opened and showed a 2–3 mm defect on the anterior aspect of the distal suture line. Prolene sutures were used to reinforce the suture line. An additional suture was utilized to reinforce the arch vessel island. The patient was weaned from CPB and had profound coagulopathy and hemorrhage, requiring blood product transfusion. The chest was left open and packed owing to coagulopathy and was successfully closed two days later. Postoperative CTA displayed decompression of local structures consistent with resolution of the pseudoaneurysm (Figure 4).

The postoperative course was complicated by the need for intermittent BiPAP for hypercarbia and hypoxia, as well as diuresis for hypervolemia. The patient progressed well and was discharged home on postoperative day 13.

### What we learned

The potential complications of ECMO may deter surgeons from its use in patients with high-risk aortic pathology who present with respiratory distress^28–31^. However, when intubation is not feasible and respiratory failure is inevitable, it can become a necessity. In this case, pseudoaneurysm repair was successful in a patient with preoperative VV ECMO, providing evidence supporting the feasibility of this technique in similarly precarious and high-risk emergencies.

This procedure carried the additional risks of a complex redo operation in a patient with a history of multiple aortic procedures. A previous case report characterized the challenges of redo operations for pseudoaneurysms, including difficulty with sternal entry that may precipitate fatal hemorrhage or air emboli.^32^ Another study noted the risk of stroke, paraplegia, or respiratory failure in redo surgeries of the ascending aorta and aortic arch.^33^ Fortunately, despite this being the patient’s third sternotomy, none of these potential risks occurred.

To our knowledge, this is the first reported case of pseudoaneurysm repair in a patient on preoperative VV ECMO. This success in the face of the extreme operative risk combining ECMO with a redo aortic operation supports the expanded use of ECMO.

### Author Statement

### Conceptualization

Jennifer S. Lawton and Hamza Aziz.

### Writing - original draft

Anson Y. Lee, Emily L. Larson, and Ifeanyi D. Chinedozi.

### Writing - review & editing

Anson Y. Lee, Emily L. Larson, Ifeanyi D. Chinedozi, Jennifer S. Lawton, and Hamza Aziz.
